# Evaluation of Serum Iron Parameters among Men Performing Regular Physical Activity—A Preliminary Study

**DOI:** 10.3390/life13030670

**Published:** 2023-03-01

**Authors:** Klaudia Zamelska, Mateusz Rzepka, Dorota Olszewska-Słonina, Alina Woźniak, Karolina Szewczyk-Golec, Iga Hołyńska-Iwan

**Affiliations:** 1 Department of Pathobiochemistry and Clinical Chemistry, Faculty of Pharmacy, Ludwik Rydygier Collegium Medicum in Bydgoszcz, Nicolaus Copernicus University, 87-100 Toruń, Poland; 2 Department of Microbiology, Faculty of Pharmacy, Ludwik Rydygier Collegium Medicum in Bydgoszcz, Nicolaus Copernicus University, 87-100 Toruń, Poland; 3 Department of Medical Biology and Biochemistry, Faculty of Medicine, Ludwik Rydygier Collegium Medicum in Bydgoszcz, Nicolaus Copernicus University, 87-100 Toruń, Poland

**Keywords:** anemia, athletes, ferritin, blood morphology

## Abstract

Iron deficiency anemia is one of the most common issues in clinical practice. It can be caused by intense physical activity, among other things. The aim of the study was to assess serum iron parameters in a group of men who engage in regular physical activity. The study group was composed of 20 men who regularly perform strength or endurance sports, whereas the control group consisted of 20 men without any sports activity. The red blood cell (RBC) parameters, platelet count (PLT), and white blood cell (WBC) count in venous blood samples were assessed with an automated hematology analyzer. The serum concentration of ferritin was determined through an immunology assay. There were no statistically significant differences between groups comparing RBC parameters and WBC. However, statistical analysis showed dissimilarity in PLT count and serum ferritin concentration comparing control and study groups (*p* < 0.05). It was shown that lower serum ferritin concentration concerns men with regular physical activity, whereas other blood parameters were not affected in this group.

## 1. Introduction

The correct state of iron metabolism depends on maintaining a strict balance between its intestinal absorption, accumulation, and release by reticuloendothelial cells and the use of this metal mainly for the synthesis of hemoglobin by cells of the erythropoietic system and in enzymatic processes and/or cell division [1,2,3,4]. These processes are regulated at the cellular and systemic level and are reflected in laboratory tests used to assess the state of iron metabolism, i.e., the concentration of iron, ferritin, transferrin, soluble transferrin receptor, and/or hepcidin [3]. Iron in the human body is distributed in two compartments, namely storage and functional. The storage compartment includes iron accumulated in liver cells and macrophages of the reticuloendothelial system, where it is associated with ferritin and hemosiderin. The functional compartment includes iron in the blood bound to transport proteins (i.e., transferrin) and iron used for the production of hemoglobin in erythroblasts and the synthesis of other ferroproteins, such as myoglobin, cytochromes, and others [1,2,3,5]. In ferroproteins, iron is bound to heme groups or iron-sulfur clusters. Apart from transporting oxygen, iron is involved in numerous enzymatic reactions as a component of enzymes or as a reaction cofactor. A particularly important function includes the involvement of ferroproteins in the processes of energy production in the mitochondria [1,2,3,6].

Specific biochemical and hematological indicators, including ferritin, transferrin, soluble transferrin receptor, and hepcidin, allow for assessing the level of iron in both compartments [3,5]. These indicators, together with the concentration of iron in the blood serum/plasma, are used to assess possible iron deficiencies in the body [4,5]. As a part of primary care, the determination of serum/plasma iron is a standard first-line test, although it has numerous limitations in the assessment of iron metabolism. Iron concentration depends on both age and sex [7]. It shows diurnal variability, with higher values observed in the morning. The concentration of iron in the blood can also be temporarily increased after eating food containing easily digestible iron and/or after supplementation [8,9]. Therefore, adequate assessment of iron metabolism is ensured by tests of the transferrin/receptor system, which supplies iron to target cells, and the determination of ferritin concentration [9].

Ferritin is found intracellularly, mainly in the cytosol, nucleus, mitochondria, and lysosomes; it is also present in a free form in the blood plasma. It consists of 24 subunits, made up of two chains: ferritin light chain (FTL) with a mass of 19 kDa and ferritin heavy chain (FTH) with a mass of 21 kDa. The combination of heterodimers gives ferritin a unique nanocage structure [6,10]. The native protein is released from the cells by the process of exocytosis. As a result, a hydrophobic space with an acidic pH of around 3.9 is formed inside the ferritin. This hydrophobic nanocage can hold up to 5000 Fe atoms. Iron enters and leaves this space by diffusion through four specific channels. Importantly, ferritin stores iron in the third oxidation state (Fe^3+^) [6,10]. The uncontrolled release of iron with the progressive degradation of ferritin underlies the process termed ferritinphagy, which initiates apoptosis by activating reactive oxygen species (ROS) and initiating lipid peroxidation [11]. Ferritin is produced mainly by the liver, muscle, heart, brain, spleen, and placenta, but also by other tissues and organs in small amounts. Different organs produce individual subunits that differ slightly in their amino acid sequence. In addition to the ferritin dimer, single chains or polymeric structures may also be present in the blood; however, they do not have an iron-binding capacity comparable to that of the ferritin dimer [6,10]. Decreased ferritin concentrations are observed in the course of iron deficiency, whereas increased levels occur during long-term inflammation and when the body is overloaded with iron, e.g., after chemotherapy [6,7,8].

The normal serum level of iron in adult men is 11–33 μmol/L (60–180 μg/dL), while iron levels in women are about 10% lower [9]. Underestimated values of iron concentration occur in states of systemic iron deficiency associated with malabsorption or coagulation disorders and in functional deficiencies caused by blocking its release from macrophages and making it available to the erythropoiesis process, e.g., in anemia of chronic diseases [4,8,12]. Iron metabolism indices are not evaluated in routine clinical practice. Patients with abnormal blood counts, especially those with reduced hemoglobin (HGB) and hematocrit (HCT) levels and characteristic symptoms of anemia, are qualified for detailed diagnostics in this regard [1,4,7,8]. Sideropenic (iron-deficiency) anemia is a common abnormality in clinical practice. The most common causes of this type of anemia include blood loss and disorders of iron absorption in the gastrointestinal tract, as well as insufficient supply of iron with food, especially during adolescence and in women of childbearing period/age. Intense physical activity may be another reason for anemia resulting from iron deficiency [13]. It has not yet been proven whether iron-related disorders associated with sports activities are solely due to increased iron loss or partly due to increased iron demand or reflect iron redistribution rather than actual iron deficiency. Irrespective of the mechanism of this phenomenon, clinical symptoms resulting from reduced iron levels occur even after a short period of mild exercise [13,14,15,16]. Due to the risk of iron metabolism disorders accompanying intense physical exercise, it should be emphasized how important it is to follow dietary recommendations regarding the amount of iron intake during exercise training in people at risk of sports anemia. Undoubtedly, in a diet to prevent iron deficiency and maintain fitness, not only should an adequate supply of iron be ensured, but also adequate levels of vitamins D, C, B12, and folic acid [2,17,18].

**Figure 1:**
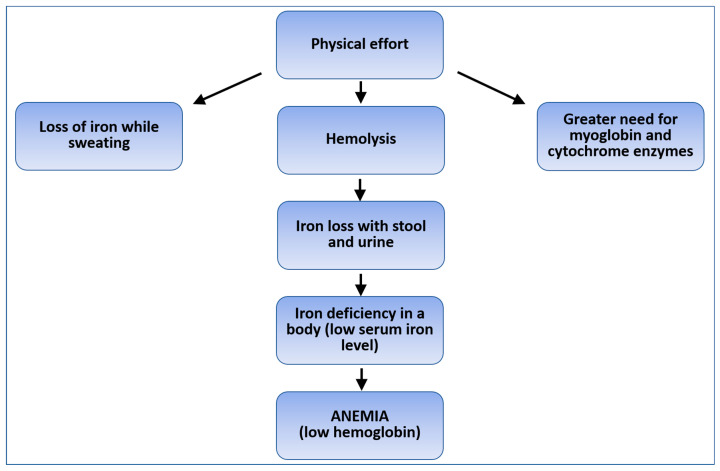
Possible mechanisms of iron loss caused by physical exertion.

Physical effort, through mechanical forces and oxidative stress, can lead to intravascular hemolysis [7]. During sports training, blood loss via the urinary tract may occur due to microscopic changes in the renal tubules caused by reduced visceral circulation. Blood loss can also occur in the gastrointestinal tract. It was found that increased intestinal movement (35%) and diarrhea (19%) often accompanied intense running, while bloody diarrhea was observed in approximately 1.2% to 2.4% of runners [14]. Despite the fact that most symptoms usually do not require immediate medical attention, gastrointestinal bleeding can limit training, degrade performance, and pose a serious danger to the athlete. Rectal bleeding is rare, but latent bleeding is present in about a quarter of marathon runners [14]. This may be due to both a reduced blood flow through the spleen caused by enhanced blood flow to the muscles and skin during prolonged exercise and an increased activity of the sympathetic nervous system that reduces blood flow through the intestine. Factors that can aggravate this condition include dehydration, overheating, hypoxia, and changes in blood composition [15]. The latent blood loss in urine and feces reflects changes in both the urinary and gastrointestinal tracts during intense physical activity. In addition, iron sequestration in macrophages reduces its absorption due to increased hepcidin production as a result of induction of the inflammatory response [16,19]. Increased pro-inflammatory markers, latent blood loss, and decreased haptoglobin levels are observed in subjects immediately after intense training, up to 24 h. Additionally, the increased loss of iron with sweat during physical effort should be noted. Figure 1 presents possible mechanisms related to iron loss following intense physical activity.

In order to better understand pathomechanisms of sports anemia and to implement appropriate diagnostic procedures, it is reasonable to accurately collect information on the above issue [13]. Thus, taking into account the current state of knowledge on the effect of physical exercise on iron metabolism, the aim of the presented study was to assess the impact of regular physical activity on the risk of developing iron deficiency anemia in healthy adult men. For this purpose, a comparison of selected iron management parameters in men with regular physical activity and a group of men not practicing sports was planned.

## 2. Materials and Methods

The research was conducted at the Department of Pathobiochemistry and Clinical Chemistry of the Ludwik Rydygier Collegium Medicum in Bydgoszcz, Nicolaus Copernicus University in Toruń, Poland. The study group consisted of 22 men (aged 22–38 years) who showed regular physical activity. Regular physical activity means moderate practice for at least 225 min a week, or at least 75 min a week when for high-intensity activity. This group was made up of men practicing endurance sports (n = 11, 55.0%) and strength sports (n = 9, 45.0%). The control group consisted of 20 men in the 21–40 age group who did not show regular physical activity (mean physical activity 35 min a week).

In addition to the frequency and type of physical activity, the criteria for inclusion in both groups were as follows: a man aged 20–40 who consented to participate in the study, of normal weight, non-smoker, using a balanced diet, denying the use of any particular type of diet, not taking any iron supplements or vitamin preparations, not suffering from chronic diseases, not taking any medications, denying any health or stress problems during the month preceding the examination and living in the same geographical region (Table 1). The listed criteria, declared by the participants and confirmed during the recruitment interview, enabled the formation of two corresponding groups differing only in physical activity. Moreover, thanks to the inclusion criteria used, other factors that could affect the studied parameters related to iron metabolism were excluded.

Venous blood samples were collected from the vein of the upper limb (around the elbow flexion) into anticoagulant (ethylenediaminetetraacetic acid, EDTA)-containing tubes for hematological tests or into tubes without anticoagulant to obtain blood serum for biochemical tests. The material was taken in the morning under standard conditions. Blood collection between 8:00 and 10:00 am from a subject in a reclining position with the arm in a horizontal plane was adopted as standard condition [12]. Red blood cell (RBC), platelet (PLT), and leukocyte (WBC) parameters were determined using the Yumizen H500 hematology analyzer (Horiba Medical, France). To obtain serum, venous blood samples collected in a tube with a coagulation activator were centrifuged at 3000 rpm for 10 min, at 18–20 °C. Serum was aliquoted (200 µL) into Eppendorf tubes and stored at −80 °C for a maximum of 3 months. The material prepared in this way was used for further analysis. Ferritin concentration in blood serum samples was measured in the next stage of the study. Before performing the analysis, the material was thawed at room temperature and then mixed using a vortex. Serum ferritin concentration was determined using a commercial enzyme immune assay (ELISA) kit (DRG Instruments, Marburg, Germany), according to the manufacturer’s protocol. The results were developed with the use of the Statistica 11.0 program, with the adopted significance level at *p* < 0.05. The statistical analysis of parametric data, including age, physical activity (minutes per week), and morphological parameters (RBC, HGB, HCT, MCV, MCH, MCHC, RDW-CV, RDV-SD, WBC, PLT), was performed with the use of Student’s *t*-test, while ferritin concentration as a non-parametric data was analyzed with the Mann–Whitney test. The effect size was calculated for ferritin and HGB concentrations, as well as RBC, HCT, MCV, MCH, MCHC, WBC, and PLT counts, with the use of the r-Pearson correlation.

The research was approved by the Bioethics Committee of the Ludwik Rydygier Collegium Medicum in Bydgoszcz Nicolaus Copernicus University in Torun (No. KB277/2018).

## 3. Results

**Figure 2:**
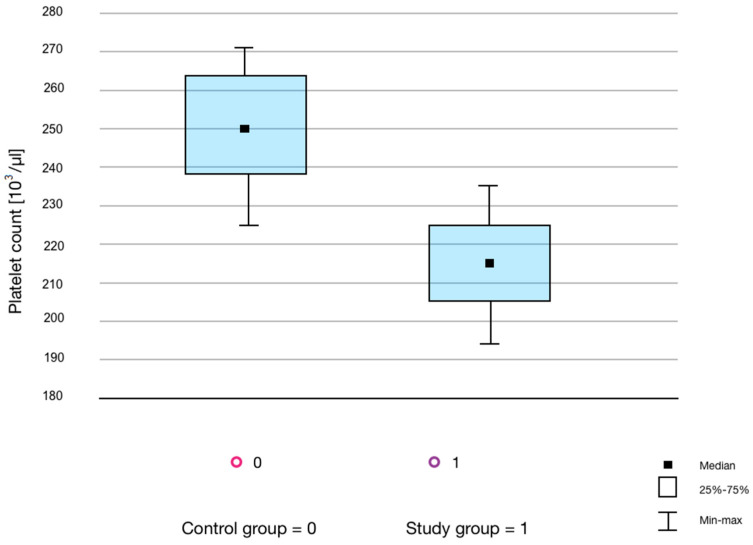
Box-plot graph showing the number of platelets in the control and study groups.

**Figure 3:**
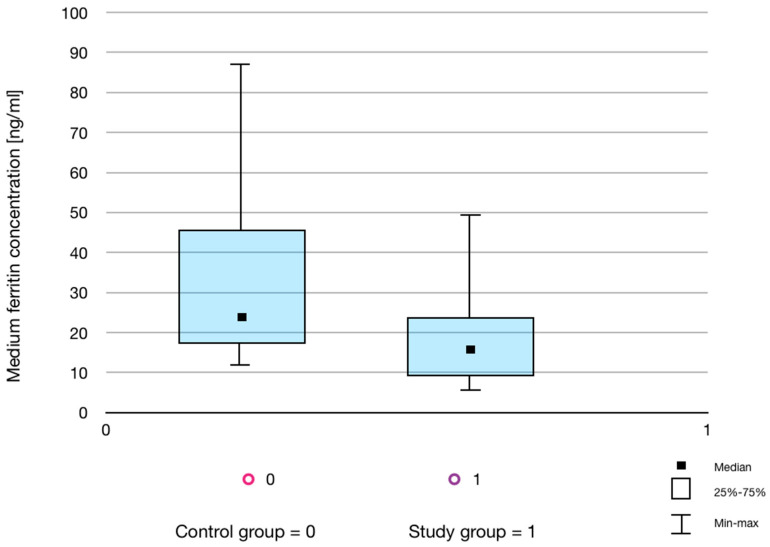
Box-plot graph showing the average ferritin concentration in the control and study groups.

Table 2 presents the results of a comparative analysis of morphology parameters and ferritin concentration in the control and the study groups. The ferritin concentration was measured in two repeats. There were no statistically significant differences in the parameters of the RBC and WBC count between the study and control groups. However, statistically significant differences in PLT count (Figure 2) and in the ferritin concentration between the groups (Figure 3) were demonstrated. No significant correlations were observed.

## 4. Discussion

Every scientific study showing that physical activity brings benefits and, when performed with caution, does not pose a health risk is important for society in the context of the obesity epidemic associated with improper diet and a sedentary lifestyle [3,7,16]. The assessment of iron metabolism and parameters related to maintaining homeostasis of oxygen distribution in the body is particularly noteworthy, as the obtained results may contribute to the spread of sports or, on the contrary, they may discourage physical activity [7,8,14].

### 4.1. Changes in Serum Ferritin Levels

A comparative assessment of ferritin concentration in the group of men showing regular physical activity compared to the group of non-training men performed in the presented study revealed statistically significant differences in mean ferritin concentrations between the examined groups. It should be emphasized that ferritin is an acute-phase protein whose concentration increases several times in the inflammation state [8,17]. However, all study participants denied any disease symptoms for several weeks prior to the examination, and none of them suffered from chronic diseases. Thus, the obtained ferritin concentrations seem to reflect the iron balance in the subjects. It should also be taken into account that the low ferritin concentrations in the training men, found in the presented study, may be at least partly due to their high outdoor activity level. Exposure to solar radiation enables the production of vitamin D in the amount appropriate for the body. It has been proven that the correct level of vitamin D in people who exercise regularly is associated with the maintenance of proper iron management and the reduction of inflammation caused by physical exercise [17]. However, in the presented study, the vitamin D level of the participants was not measured. Results similar to the presented research were obtained in the study of Henningar et al. [18], in which the effect of intensive training on the hepcidin, ferritin and HGB concentrations, RBC count, and total iron binding capacity in men over a 72 h period was assessed. A reduction in ferritin levels and an increase in hepcidin by as much as 50% were observed and remained at this level until the intense exercise.

The concentration of ferritin measured in the blood reflects the concentration of intracellular ferritin. Intracellular ferritin is the main protein that stores and makes iron available for cellular metabolism. Therefore, the measurement of its blood plasma concentration translates into an assessment of iron utilization by cells [20]. Cells that require iron exhibit ferritin receptors on their surface. Erythroblasts are the cells with the largest number of plasma membrane ferritin receptors. In addition, a number of intracellular receptors result in an increased demand for iron by cells and individual cellular organelles [21]. These intracellular receptors include, among others, the nuclear androgen receptor, whose activity is regulated by iron ions. The importance of ferritin for iron availability makes its measurement one of the tests recommended for athletes to assess iron metabolism. Nabhana et al. [22] showed that increased demand for iron and increased iron loss are associated with the development of iron deficiency. The first parameter to decrease in this condition is plasma ferritin, followed by a decrease in hemoglobin and hematocrit. Ultimately, the authors recommended the determination of ferritin concentration as a screening for anemia in professional athletes because normal ferritin values indicate suboptimal iron status and indicate the appropriate adjustment of iron demand in relation to training and diet [22]. The measurement of ferritin concentration is a test that allows the assessment of a number of processes and pathomechanisms taking place in the body [20,21,23,24]. Morwald et al. [23] proved that high levels of ferritin are strongly correlated with the development of inflammation in the course of metabolic syndrome and obesity due to an increased iron uptake by hepatocytes and adipocytes. Together with elevated alanine aminotransferase (ALT) and aspartate aminotransferase (AST) activities, an increase in ferritin may indicate progressive fatty liver disease [23]. Strong correlations between obesity in adolescents and elevated ferritin levels were also described [24]. Interestingly, in the course of COVID-19, high ferritin levels correlated with a more frequent occurrence of cytokine storm in patients [25].

### 4.2. Changes in Red Blood Cell Parameters

Anemia is defined as a reduced HGB concentration, RBC number, and/or HCT in a venous blood sample [5,8,9]. This definition ignores the fact that anemia is a real reduction in the body’s total HGB mass (absolute anemia). In contrast, pseudoanemia is characterized by a reduced HCT value and HGB concentration due to clearly increased plasma volume, with normal RBC count and total HGB mass. Other blood parameters, in particular ferritin, mean corpuscular volume (MCV), and mean corpuscular hemoglobin (MCH), are usually within the normal range. The increase in plasma volume in athletes can be significant and results from repeated training cycles over the year. Its size depends on the intensity and duration of physical exertion. It is estimated that in about 10–15% of athletes, mainly endurance athletes, diluting pseudoanemia can be observed, especially when the training time exceeds 10 h a week [26]. HGB concentration with other indicators of the RBC system belongs to commonly performed diagnostic tests in people showing physical activity. The results of other studies showed that the mass of HGB in endurance athletes is much higher than in people with a low level of fitness [16]. HGB concentration in these athletes, based on the mass of the body, can be up to 40–50% higher than in untrained people. These results also indicated that the mass of HGB differs in athletes depending on the specifics of the sport [27].

In the presented research comparing the RBC parameters, no statistically significant differences were found between the study and control groups. It should be taken into consideration, however, that the indices dependent on the blood dilution (HGB, RBC, and HCT) may have affected the results [27]. Similarly, in the research conducted by Bachero-Mena et al. [28], no statistically significant differences were found between the blood parameters related to oxygen transport, such as the number of RBC, HGB concentration, and MCH value, and RBC distribution width (RDW). In similar studies on long-term training subjects, Lai et al. [29] also did not show any changes or showed insignificant changes in HGB concentrations, but they observed changes in MCV values. However, long-term studies of recreational athletes practicing endurance sports, carried out by Schmidt et al. [30], showed a 6% increase in HGB. These results may suggest that changes in HGB concentration and RBC count are the result of long-term training. Based on our own results and those available in the literature, it can be concluded that the impact of training on the values of RBC indices has not yet been fully characterized.

It should be noted that the values of parameters of the red cell system, including RBC, HGB, RDW, and ferritin, are also related to proper nutrition [3,4,8]. The use of a balanced diet in combination with physical activity seems to be of particular importance for maintaining systemic iron homeostasis [7,9,13]. In the course of the presented study, all participants were asked about their diet. Each of them claimed that they did not follow a special diet, only a balanced one. In addition, the participants from both studied groups denied the use of any dietary supplements. It follows that both groups, namely the men performing regular physical exercise and men who did not practice sports, were homogeneous in terms of diet. Therefore, the values of the above-mentioned parameters found in the presented study could reflect the level of physical activity of the subjects studied. However, consistent results of these parameters in both examined groups might indicate a greater importance of diet than regular moderate physical exercise on the red blood cell system.

### 4.3. Changes in the WBC Count

Considering the assessment of the WBC count, there were no statistically significant differences in the count of leukocytes between the examined groups in the presented study. On the other hand, the research conducted by Horn et al. [31] among 2247 women and men showed a significantly lower total count of WBC, including neutrophiles, in people practicing aerobic sports. Lower leukocyte value in trainers could probably be the result of the adaptive response of the body. A lower WBC count among endurance trainers compared to team sports and control groups has also been reported in many other studies [27,28,32]. Different types and intensities of physical activity, and also the small study group examined in the presented research could explain these discrepancies. In the presented research model, blood samples were collected under standard conditions, i.e., on an empty stomach, after an overnight rest, and, importantly, the participants were asked to refrain from training or to perform only low-intensity training on the day preceding the study. It seems that ensuring rest may be essential for the WBC count. Rest allows the organism to maintain a balance between the amount of WBC in the plasma and inter-tissue spaces, especially in the case of micro-damage to muscles caused by physical effort [30]. It could be assumed that, in the examined subjects, the exercise performed did not cause such muscle damage that would be responsible for the initiation of the inflammatory state and, consequently, would lead to permanent changes in the number of WBC and ferritin concentrations.

### 4.4. Changes in the PLT Count

In the presented study, statistically significant differences were observed in the PLT count between the study and control groups. It is worth noting that, despite statistically significant differences in the number of PLTs, these values were within the reference range for both groups. The main factor influencing the reduction of the PLT amount is their consumption in the course of thrombotic events [3,5,33]. In the presented research model, the participants from both the training and control groups did not suffer any injuries indicating bleeding several weeks before blood sampling. In addition, the onset of inflammation was not demonstrated in the training people; therefore, it is unlikely that the lower amount of PLTs observed in this study group was due to lower PLT production in the course of chronic inflammation. Results opposite to the presented study were obtained by Davis et al. [34], assessing the impact of physical effort and physical condition on the function and number of PLTs. An increase in thrombocyte count after 6 and 12 weeks of regular, controlled training compared to non-exercisers was shown in their study. Heber et al. [33] also showed that intensive exercise caused a temporary increase in the number of PLTs, and this increase was mainly due to the hemoconcentration and release of thrombocytes from the liver, lungs, and spleen. It seems that in the examined people with regular physical activity, rest could prevent the mechanisms of PLT release from tissues observed by other authors [33,34].

### 4.5. Goals and Weaknesses of the Experiment

The advantage of the presented study was the assessment of ferritin concentration and morphological parameters in the participants, who represented a homogeneous group in terms of sex, age, and a number of factors that could significantly affect iron metabolism. Only healthy men, non-smokers, following a standard diet, not using iron supplements, vitamin preparations, or any medications chronically or for 4 weeks before the study were included in the study. The only difference was the intensity of physical activity (see Table 1, Student’s *t*-test, *p* = 0.00089). The homogeneity of both the study and control groups was reflected in the homogeneous results of morphology and ferritin concentration (see Table 2, Figure 1 and Figure 3). It should be emphasized that Nabhana et al. [22], in their studies, recommended the use of ferritin tests to assess the body’s efficiency and early detection of iron deficiency anemia.

Speculations regarding the results are quite problematic due to the small group of subjects participating in the study. People included in the study group showed various levels of physical activity. Certainly, the lack of division into appropriate disciplines is a limitation of this study. The fact that the test material was collected once is the next limitation of the study. Therefore, future studies should consider a higher number of measurements in a specified period of time for a more thorough analysis of the impact of training loads on the physiological adaptations of the body. An additional limitation includes a limited number of measured laboratory parameters that could be used to assess the causes and/or degree of anemia. The authors plan to extend both the number of tested people and the number of laboratory parameters, including ferritin chains, a soluble transferrin receptor, as well as vitamin D concentration.

## 5. Conclusions

Due to the growing epidemic of obesity, conducting research aimed at explaining the changes in the course of metabolism occurring during regular physical activity is of great importance to society. According to the results of the presented study, regular physical activity did not affect the number of erythrocytes and other measured red blood cell parameters, as well as the white blood cell count. However, a decrease in the number of platelets in the group of people showing regular physical activity was found. In addition, the concentration of ferritin, an acute phase protein, which is a parameter used to assess the transport and utilization of iron in the body, was lower in the training group compared to the group of men who did not exercise regularly. The results obtained may indicate a reduction in the level of inflammation in the body due to regular exercise. Importantly, no tendency to negative changes in iron metabolism or the development of anemia in people regularly training was shown in the presented experimental model.

## Figures and Tables

**Table 1:** Participant demographics.

	Control	Sportsmen	*p*
Age (years) mean (minimum–maximum)	25 (21–40)	27 (22–38)	0.132
Physical activity (minutes/week) mean (minimum–maximum)	35 (20–50)	225 (180–360)	0.00089
Body mass index (kg/m^2^)	18.5–24.9		
Iron supplementation	no		
Diet	normal		
Smoking	no		
Acute/Chronic diseases	no		
Medicaments	no		
Stress	no		
Well-condition	yes		

Captions: the results of Student’s *t*-test with *p* < 0.05.

**Table 2:** Test results of measured parameters related to iron metabolism in the examined and control groups.

Parameter	Control Group (n = 20)		Study Group (n = 20)			
	Median	Lower-Upper Quartile	Median	Lower-Upper Quartile	*p*	r-Pearson
RBC [10^6^/µL]	5.08	4.91–5.45	5.17	4.87–5.31	0.79	−0.36
HGB [g/dL]	15.90	15.40–16.55	15.55	15.00–15.85	0.18	−0.35558
HCT [%]	47.00	44.90–49.85	46.75	44.55–47.60	0.41	−0.3342
MCV [µm^3^]	91.00	88.20–94.00	90.55	89.00–92.10	0.46	0.2891
MCH [pg]	30.90	29.50–31.50	30.10	29.80–30.55	0.27	−0.1639
MCHC [g/dL]	33.60	32.85–34.30	33.40	32.90–33.75	0.33	0.1433
RDW-CV [%]	13.10	12.80–13.65	13.60	12.95–13.95	0.09	-
RDW-SD [µm^3^]	40.30	39.50–43.70	41.20	40.30–44.10	0.59	-
PLT [10^3^/µL]	245.50	212.00–283.50	217	195.50–250.50	0.04	0.0403
WBC [10^3^/µL]	5.63	5.20–7.56	5.70	4.87–6.51	0.31	−0.1515
Ferritin—measurement 1 [ng/mL]	21.53	16.40–39.53	14.87	9.19–22.24	-	
Ferritin—measurement 2 [ng/mL]	25.00	17.65–52.78	16.74	9.36–22.18	-	
Ferritin—average [ng/mL]	23.12	17.44–46.16	15.81	9.26–22.69	0.01	−0.0856

Abbreviations: n—group size, HCT—hematocrit, HGB—hemoglobin concentration, MCH—mean corpuscular hemoglobin, MCHC—mean corpuscular hemoglobin concentration, MCV—mean corpuscular volume, PLT—platelet count, RBC—red blood cell count, RDW-CV—red blood cell distribution width, coefficient of variation, RDW-SD—red blood cell distribution width, standard deviation, WBC—white blood cells count, *p*—level of significance.

## Data Availability

Data will be able on request: igaholynsk@cm.umk.pl.
